# Comparison of enhanced laparoscopic imaging techniques in endometriosis surgery: a diagnostic accuracy study

**DOI:** 10.1007/s00464-019-06736-8

**Published:** 2019-04-26

**Authors:** Marit C. I. Lier, Stijn L. Vlek, Marjolein Ankersmit, Peter M. van de Ven, Judith J. M. L. Dekker, Maaike C. G. Bleeker, Velja Mijatovic, Jurriaan B. Tuynman

**Affiliations:** 1 Department of Reproductive Medicine, Endometriosis Center, Amsterdam UMC—Location VUmc, Amsterdam, The Netherlands; 2 Department of Surgery, Endometriosis Center, Amsterdam UMC—Location VUmc, De Boelelaan 1118, ZH7F20, 1081HZ, Amsterdam, The Netherlands; 3 Department of Epidemiology and Biostatistics, Amsterdam UMC—Location VUmc, Amsterdam, The Netherlands; 4 Department of Pathology, Endometriosis Center, Amsterdam UMC—Location VUmc, Amsterdam, The Netherlands

**Keywords:** Endometriosis, Laparoscopy, Imaging techniques, 3D, NBI, Fluorescence

## Abstract

**Background:**

For surgical endometriosis, treatment key is to properly identify the peritoneal lesions. The aim of this clinical study was to investigate if advanced imaging improves the detection rate by comparing narrow-band imaging (NBI), near-infrared imaging with indocyanine green (NIR-ICG), or three-dimensional white-light imaging (3D), to conventional two-dimensional white-light imaging (2D) for the detection of peritoneal endometriotic lesions.

**Methods:**

This study was a prospective, single-center, randomized within-subject, clinical trial. The trial was conducted at Amsterdam UMC—Location VUmc, a tertiary referral hospital for endometriosis. 20 patients with ASRM stage III–IV endometriosis, scheduled for elective laparoscopic treatment of their endometriosis, were included. During laparoscopy, the pelvic region was systematically inspected with conventional 2D white-light imaging followed by inspection with NBI, NIR-ICG, and 3D imaging in a randomized order. Suspected endometriotic lesions and control biopsies of presumably healthy peritoneum were taken for histological examination. The pathologist was blinded for the method of laparoscopic detection. Sensitivity and specificity rates of the enhanced imaging techniques were analyzed. McNemar’s test was used to compare sensitivity to 2D white-light imaging and Method of Tango to assess non-inferiority of specificity.

**Results:**

In total, 180 biopsies were taken (117 biopsies from lesions suspected for endometriosis; 63 control biopsies). 3D showed a significantly improved sensitivity rate (83.5% vs. 75.8%, *p* = 0.016) and a non-inferior specificity rate (82.4% vs. 84.7%, *p* = 0.009) when compared to 2D white-light imaging. The single use of NBI or NIR-ICG showed no improvement in the detection of endometriosis. Combining the results of 3D and NBI resulted in a sensitivity rate of 91.2% (*p* < 0.001).

**Conclusion:**

Enhanced laparoscopic imaging with 3D white light, combined with NBI, improves the detection rate of peritoneal endometriosis when compared to conventional 2D white-light imaging. The use of these imaging techniques enables a more complete laparoscopic resection of endometriosis.

Endometriosis, characterized by endometrial deposits in the peritoneal cavity, is a benign but chronic and potentially harmful disorder associated with pelvic pain complaints, subfertility, and/or pelvic organ dysfunction [1]. Although endometriosis can be suspected on presenting symptoms, physical examination and imaging [e.g., transvaginal ultrasound (TVU) and magnetic resonance imaging (MRI)], laparoscopic identification, and histological verification of endometriotic tissue remain the gold standard for the final diagnosis of the disease [2]. Treatment of symptomatic endometriosis, especially when not responding to hormonal treatment, consists of laparoscopic excision or ablation of endometriotic tissue [2]. However, the identification of endometriotic tissue during laparoscopy is not always clear which may partly contribute to the high rates of recurrence reported after surgical treatment (40–50% at 5 years) [3]. The polymorphic appearance of endometriotic lesions is supposed to be the origin of this impaired visual diagnosis during laparoscopy, especially non-pigmented endometriotic lesions which are hard to distinguish from healthy peritoneal tissue [4–6]. Previous studies reported a positive predictive value of 2D white-light laparoscopy of only 66% [7]. Therefore, complete resection of endometriosis is difficult and re-operation, due to symptomatic recurrence, occurs in more than 50% of the patients [8]. The high costs of re-operation and the associated morbidity emphasize the importance of a more complete resection during primary surgery.

Enhanced laparoscopic imaging techniques, already frequently used in other fields of surgery [9–12], are promising additives to the intra-operative detection of endometriosis [13]. These techniques visualize differences in (neo)vascularization and epithelial thickness, possibly enabling an improved detection of endometriosis. Previous studies showed that with the use of 5-aminolevulinic acid-induced fluorescence (5-ALA) [14–16], autofluorescence imaging (AFI) [17–19], and narrow-band imaging (NBI) [20, 21], endometriotic lesions were detected with a better sensitivity and equal specificity compared to 2D white-light imaging. Moreover, with these techniques additional endometriotic lesions were found that were not visualized with 2D white-light imaging only. The 5-ALA technique was, however, difficult to implement due to high costs and patient side effects, such as postoperative photosensitivity [14–16]. NBI has shown to be a more feasible technique available in most laparoscopic platforms and without any side effects. The effectiveness for the detection of peritoneal endometriosis of more recently introduced laparoscopic imaging techniques, such as indocyanine green near-infrared imaging (ICG-NIR) [22, 23] or three-dimensional laparoscopy (3D), has not yet been investigated.

To directly compare these different enhanced laparoscopic imaging techniques, a clinical trial was conducted. We aimed to investigate which enhanced laparoscopic imaging technique is the most accurate for the detection of peritoneal endometriotic lesions, with respect to the sensitivity and specificity of the investigated techniques and compared to 2D white-light imaging.

## Materials and methods

### Study design

This study was a prospective, single-center, randomized within-subject, clinical trial conducted at the Amsterdam UMC—Location VUmc (VUmc, Amsterdam, the Netherlands). The study was approved by the institutional review board of the VUmc (METC VUmc 2015.392) and was registered as the LITE study (Laparoscopic Imaging Techniques in Endometriosis therapy) in the Dutch Trial Register (NTR5614, 06 January 2016). All participants provided informed consent prior to participation.

### Patients

Women with moderate to severe endometriosis (American Society for Reproductive Medicine (ASRM), stage III or IV), who had an indication for elective laparoscopic treatment were asked for participation and informed consent. Endometriosis had to be previously surgically confirmed or likely to be present based on TVU or MRI findings (including uni- or bilateral ovarian endometrioma). All eligible patients were approached during the study period. Women were included if they were aged over 18 years and were premenopausal. Excluded from participation were women who were legally or mentally incapable or unable to give informed consent; pregnant women; women who had an American Society of Anesthesiologists (ASA) score > 3; major open abdominal surgery in the past; a known malignancy; an iodine allergy or hypersensitivity reaction to prior usage of ICG; use of any medication with a known interaction with ICG; chronic kidney failure (eGFR < 55); or abnormal liver enzyme tests (ASAT, ALAT, AF, and yGT > 2 times the maximum normal value).

### Techniques

In this trial, the following laparoscopic imaging techniques were studied: narrow-band imaging (NBI), near-infrared imaging with indocyanine green (NIR-ICG), and three-dimensional white-light imaging (3D). These techniques were compared to conventional two-dimensional high-definition white-light imaging (2D) using the laparoscopic system CLV-180 EVIS EXERA II platform (Olympus, Center Valley, USA) with an ENDOEYE FLEX 3D deflectable videoscope in the 2D viewing mode.

#### Narrow-band imaging (NBI)

NBI was applied using the standard available settings from the laparoscopic system CLV-180 EVIS EXERA II platform (Olympus, Center Valley, USA) with an ENDOEYE FLEX 3D deflectable videoscope. In NBI imaging, two specific wavelengths of light are filtered that are strongly absorbed by hemoglobin. The shorter wavelength (415 nm) only penetrates the superficial layers of the mucosa and is absorbed by capillary vessels in the surface of the mucosa. The second wavelength (540 nm) penetrates deeper and is absorbed by blood vessels located deeper within the mucosal layer, allowing visualization of the vasculature of suspected endometriotic lesions.

#### Near-infrared imaging with indocyanine green (NIR-ICG)

For the NIR-ICG procedure indocyanine green (ICG, ICG-PULSION®, PULSION Medical Systems AG, Munich, Germany) was intravenously applied through a peripheral infusion during surgery. ICG is a sterile water soluble tricarbocyanine dye with a peak spectral absorption in blood (plasma) at 800–810 nm (range: 750–950 nm). ICG powder was diluted with sterile water for injection to a final solution of 0.25 mg/mL. A bolus of 1 mL ICG solution was injected through a peripheral IV line at the start of the procedure. If visualization was inadequate, an extra bolus of 1 mL was administered. ICG was administered directly under near-infrared vision, and after administration a time period of 5 min was used for the detection of lesions. The Olympus VISERA Pro (Olympus, Center Valley, USA) laparoscopic platform was used with a modified filter on the videoscope that excited near-infrared light (800 nm) and filtered near-infrared reflection in the range of 700–900 nm, enabling visualization of the increased (neo)vascularity of endometriotic lesions.

#### Three-dimensional white-light imaging (3D)

3D imaging was applied using the laparoscopic system CLV-180 EVIS EXERA II platform (Olympus, Center Valley, USA) with an ENDOEYE FLEX 3D deflectable videoscope. The dual lens generated 3D images, projected on a 3D monitor. Lightweight polarized 3D eyewear integrated the images into 3D vision and enabled depth perception with accurate color reproduction.

### Surgery and imaging

**Fig. 1:**
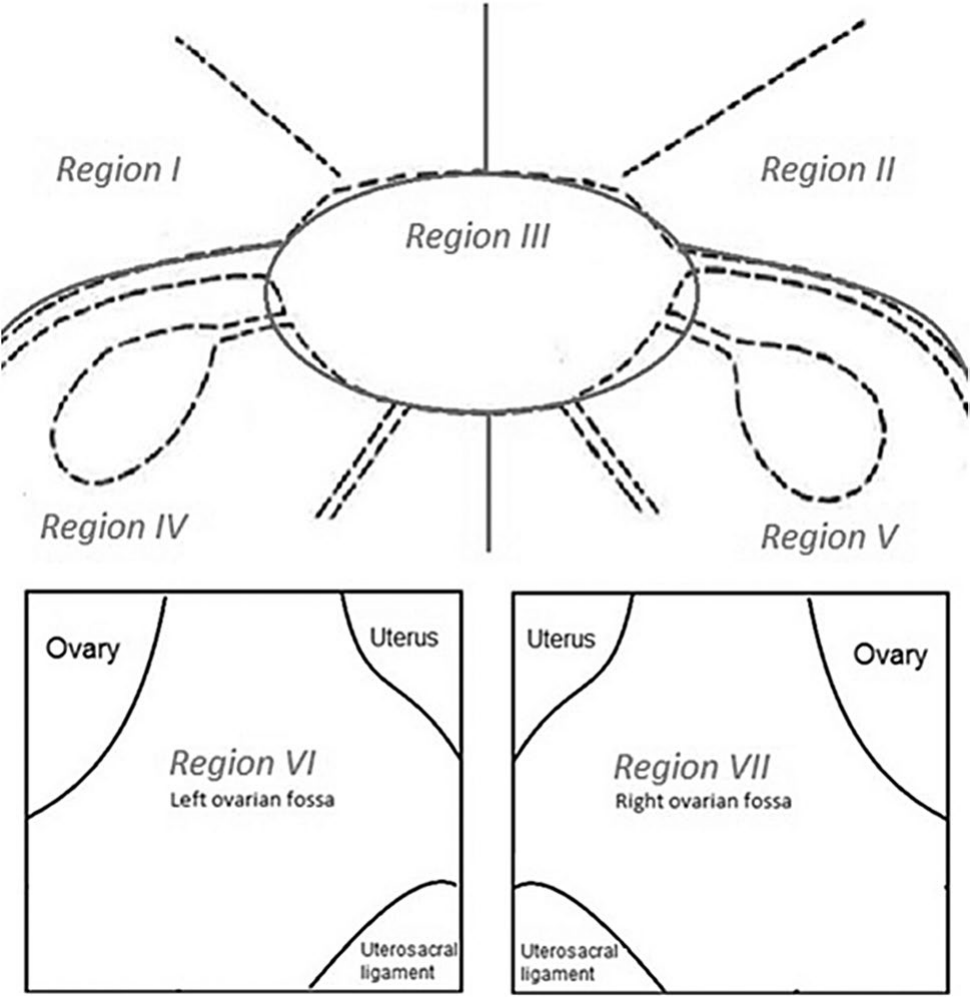
Schematic image of pelvic region

Patients were placed in the supine position and received general anesthesia. After introduction of the videoscope through a 12-mm trocar, the abdominal cavity was inspected. Two additional 5-mm trocars were placed to facilitate exposure of the pelvic cavity. For a systematic approach and recording of the suspected lesions, the pelvic cavity was divided in seven regions: *region I*—left lateral pelvic wall and bladder peritoneum; *region II—*right lateral pelvic wall and bladder peritoneum; *region III—*peritoneal surface of the uterus; *region IV—*left lower region of the pelvis (including ovarian surface, round ligament, uterosacral ligament, and Douglas’ Pouch); *region V—*right lower region of the pelvis (including ovarian surface, round ligament, uterosacral ligament, and Douglas’ Pouch); *region VI—*left ovarian fossa and *region VII—*right ovarian fossa [Fig. 1]. All regions were first inspected systematically with conventional 2D white-light imaging. Inspection was repeated with the other imaging techniques (NBI, NIR-ICG, 3D), applied in a randomized order, generated by online randomization tool Sealed Envelope Ltd. (London, United Kingdom). Visual diagnosis of endometriosis was confirmed according to previously described criteria [24, 25]. All inspections were performed by the same operating physician (VM). Suspected endometriotic lesions were identified, documented, and numbered. After full surveillance with all imaging techniques and documentation, all suspected endometriotic lesions were excised. Biopsies of healthy appearing peritoneum were taken as negative controls. No biopsies were taken of regions with increased risk of iatrogenic damage, bleeding, or perforation, such as the intestines or lesions nearby large blood vessels.

The collected specimens were processed, hematoxylin and eosin (H&E) stained, and assessed upon the presence of endometriosis (identification of endometriotic glands and/or endometrial stroma). Additional immunostaining for CD10 and estrogen receptors (ER) was applied if the presence or absence of endometriosis could not be confirmed based on the H&E staining only [26–28]. The pathologist was blinded for the method of detection (e.g., the used imaging technique) and for the clinical suspicion of endometriosis (e.g., specimens obtained from areas suspected or unsuspected (control) for endometriosis).

### Outcomes

The primary objective was to evaluate the sensitivity and specificity rates of the imaging techniques in the detection of peritoneal endometriosis, when compared to 2D white-light imaging. Secondary outcomes were false-negative rate, false-positive rate, and accuracy.

### Sample size calculation

Sample size calculation for this study was based on the number of endometriotic lesions needed to achieve 80% power to detect an increase in sensitivity from 72% (for conventional 2D white light) to 85% (for enhanced imaging techniques) assuming 70% of the endometriotic lesions to be detected by all techniques. Sample size was based on a two-sided McNemar test. Power analysis performed in PASS revealed a required number of 81 pathologically confirmed endometriosis lesions. We expected to find a mean number of 5.5 endometriotic lesions per patient in our population as previously described [29]. Therefore, the sample size was set at 20 patients.

### Statistics

After final pathologic results were confirmed, the outcomes were put in two-by-two contingency tables to calculate the primary outcomes: sensitivity [true positives/(true positives + false negatives)] and specificity [true negatives/(true negatives + false positives)] rates for each imaging technique. Also the false-negative rates [false negatives/(true positives + false negatives)] and false-positive rates [false positives/(true negatives + false positives)] were calculated. Test accuracy was calculated for this specific set of biopsies [(true positives + true negatives)/total biopsies]. Area under the receiver operator characteristics (AUROC) was calculated for each imaging technique. Comparative testing was only performed for the primary outcomes (sensitivity and specificity). To compare sensitivity of the enhanced imaging techniques (3D, NBI, and ICG) to conventional 2D white-light imaging, McNemar’s test was used. Results were considered statistically significant with *p* < 0.05 for two-sided testing. Non-inferiority of specificity of enhanced imaging techniques was tested using the method of Tango [30] assuming the prespecified non-inferiority margin of 10%. For non-inferiority a one-sided *p* < 0.025 was considered significant. Additional analyses were performed where we took into account clustering of lesions within patients. Sensitivity was compared using Generalized Estimating Equations (GEE) with an exchangeable working correlation structure to account for within-patient correlation of test outcomes. To assess non-inferiority of specificity, a similar approach was used. From the GEE output, a *p* value for non-inferiority was derived from the estimate of the difference in specificities and the reported standard error of this difference where we assumed that the estimated difference was normally distributed. Post hoc analyses were performed in which we evaluated the sensitivity and specificity for combining NBI and 3D where positivity was defined as being either positive on at least one (NBI and/or 3D) of these methods or positive on both (NBI and 3D) methods, respectively. Statistical analyses were performed with SPSS 22 (IBM, Chicago, USA) software. The non-inferiority tests assuming independence of observations were performed using Excel.

## Results

### Inclusions

Between February 2016 and May 2017, 20 participants were included in this trial. Baseline characteristics are shown in [Table 1]. In total, 180 biopsies were taken, of which 117 biopsies were taken from lesions suspected for endometriosis and 63 biopsies were taken from presumably healthy peritoneum (negative controls). The median number of endometriotic lesions per patient was 5.5 (IQR 5–7.5) with a median number of 3.5 control biopsies (IQR 2–4). After histological examination, endometriosis was confirmed in 91 biopsies, and 85 biopsies were negative for endometriosis. Four biopsies were inconclusive, meaning that the presence or absence of endometriosis could not be confirmed after histological examination.

**Table 1:** Baseline characteristics

		Total (*n* = 20)
Age at surgery (yrs) *Median (IQR)*		34.5 (29.3–39.5)
Race *n (%)*	Caucasian	17 (85%)
BMI (kg/m^2^)	< 25	12 (60%)
*n (%)*	25–30	8 (40%)
Parity *Median (IQR)*		0 (0–1)
Active childwish *n (%)*		7 (35%)
Prior abdominal surgery *n (%)*		8 (40%)
Reported pre-operative complaints *n (%)*	Dysmenorrhea	19 (95%)
	Dyschezia	13 (65%)
	Dysuria	1 (5%)
	Dyspareunia	10 (50%)
Use of pre-operative medication *n (%)*	Yes, oral contraceptives	8 (40%)
	Yes, GnRH agonist	3 (15%)
	No	9 (45%)

*BMI* body mass index, *IQR* interquartile range, *n* number, *yrs* years

### Imaging techniques

Biopsies with inconclusive results were excluded when calculating and comparing the diagnostic properties of the imaging techniques. Table 2 displays the primary outcomes, sensitivity and specificity rates of the different imaging techniques. 3D white-light imaging showed a significantly improved sensitivity rate [83.5% vs. 75.8%, *p* = 0.016 (uncorrected)/*p* = 0.005 (multi-level)] and a non-inferior specificity rate [82.4% vs. 84.7%, *p* = 0.009 (uncorrected)/*p* = < 0.001 (multi-level)] when compared to 2D white-light imaging. The single use of NBI or NIR-ICG showed no improvement in the detection of endometriosis. False-positive rates were 15.3%, 17.6%, 29.4%, and 11.0%; false-negative rates (i.e., miss rates) were 24.2%, 16.5%, 18.7%, and 63.9%; accuracy was 80.1%, 83.0%, 76.1%, and 58.5% for 2D white-light imaging, 3D white-light imaging, NBI, and NIR-ICG, respectively. Combining the results of 3D white-light imaging and NBI in a post hoc analysis resulted in a significantly improved sensitivity rate for the detection of endometriotic lesions [91.2% vs. 75.8, *p* < 0.001 (uncorrected)/p < 0.001 (multi-level)], however with a specificity rate inferior to 2D white-light imaging [0.6% vs. 75.8%, *p* = 0.90 (uncorrected)/*p* = 0.83 (multi-level)] [Table 3].

**Table 2:** Sensitivity and specificity rates

Visual examination and endometriosis suspected based on		Histological examination and presence of endometriosis confirmed				*p* Value (uncorrected/multi-level)
		Yes	No			
2D (WL)	Yes	69	13	Sensitivity	75.8%	–
*(n* = *176)*	No	22	72	Specificity	84.7%	–
				FP-rate	15.3%	
				FN-rate	24.2%	
				Accuracy	80.1%	
NBI	Yes	74	25	Sensitivity	81.3%	*p* = 0.36/*p* = 0.29
*(n* = *176)*	No	17	60	Specificity	70.6%	*p* = 0.90/*p* = 0.83
				FP-rate	29.4%	
				FN-rate	18.7%	
				Accuracy	76.1%	
NIR-ICG	Yes	30	9	Sensitivity	36.1%	*p* < 0.001/*p* < 0.001^a^
*(n* = *165)*	No	53	73	Specificity	89.0%	*p* = 0.002/*p* < 0.001^b^
				FP-rate	11.0%	
				FN-rate	63.9%	
				Accuracy	58.5%	
3D	Yes	76	15	Sensitivity	83.5%	*p* = 0.016/*p* = 0.005^a^
*(n* = *176)*	No	15	70	Specificity	82.4%	*p* = 0.009/*p* = < 0.001^b^
				FP-rate	17.6%	
				FN-rate	16.5%	
				Accuracy	83.0%	

*2D* 2D imaging, *3D* 3D imaging, *FN-rate* false-negative rate, *FP-rate* false-positive rate, *NBI* narrow-band imaging, *NIR-ICG* near-infrared imaging with indocyanine green, *n* number, *WL* white-light imaging

^a^*p* < 0.05 for the McNemar’s test for differences in sensitivity

^b^*p* < 0.025 for establishing non-inferiority of specificity compared to WL

**Table 3:** Sensitivity and specificity rates for combination 3D and/or NBI

Visual examination and endometriosis suspected based on		Histological examination and presence of endometriosis confirmed				*p* Value (uncorrected/ multi-level)
		Yes	No			
3D AND NBI	Yes	67	15	Sensitivity	73.6%	*p* = 0.77/*p* = 0.83
*(n* = *176)*	No	24	70	Specificity	82.4%	*p* = 0.009/*p* < 0.001^a^
				FP-rate	17.6%	
				FN-rate	26.4%	
				Accuracy	77.8%	
3D AND/OR NBI	Yes	83	25	Sensitivity	91.2%	*p* < 0.001/*p* < 0.001^b^
*(n* = *176)*	No	8	60	Specificity	70.6%	*P* = 0.90/*p* = 0.38
				FP-rate	29.4%	
				FN-rate	8.6%	
				Accuracy	81.3%	

3D AND NBI displays that endometriosis was suspected upon visual inspection of both 3D and NBI; 3D AND/OR NBI displays that endometriosis was suspected upon either 3D, NBI, or both techniques

*3D* 3D imaging, *FN-rate* false-negative rate, *FP-rate* false-positive rate, *NBI* narrow-band imaging, *n* number

^a^*P* < 0.025 for establishing non-inferiority of specificity compared to WL

^b^*P* < 0.05 for the McNemar’s test for differences in sensitivity

**Fig. 2:**
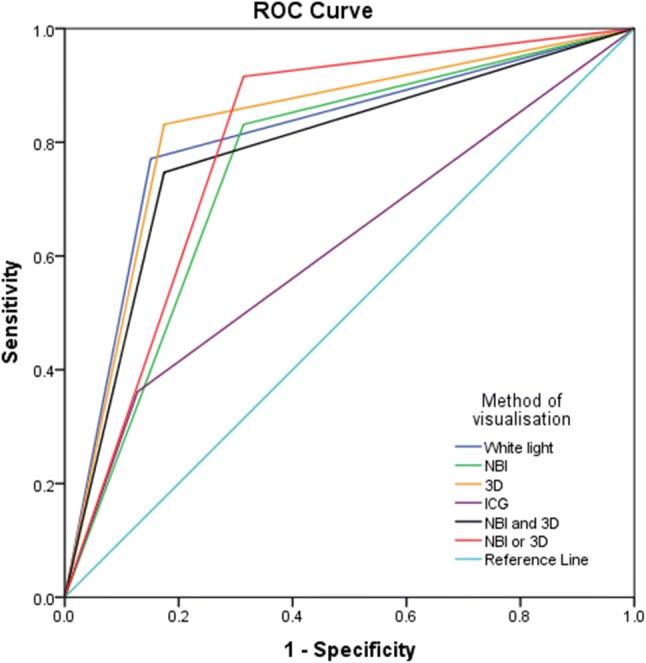
AUROC curves

For all imaging techniques and the post hoc analysis for combining 3D and NBI, AUROC has been generated [Table 4] [Fig. 2].

**Table 4:** Area under the receiver operator characteristics

Imaging technique	Area
2D (WL)	.810
NBI	.759
NIR-ICG	.617
3D	.828
Post hoc analysis	Area
NBI AND 3D	.786
NBI AND/OR 3D	.801

*2D* 2D imaging, *3D* 3D imaging, *NBI* narrow-band imaging, *NIR-ICG* near-infrared imaging with indocyanine green, *WL* white-light imaging

Twenty-two biopsies were positive for endometriosis after histological examination, but were not visualized with 2D white-light imaging; of these biopsies, 12/22 were visualized with NBI, 4/22 with NIR-ICG, and 7/22 with 3D white-light imaging. 7/22 biopsies were not visualized with any of the enhanced imaging techniques or conventional 2D white-light imaging.

### Intra-operative parameters

Operating time was extended with a median of 30 min (30–37.5 min) due to the thorough inspection of the peritoneum with all the imaging techniques including the histological sampling. The median amount of blood loss was 50 mL (IQR: 27.5 mL–100 mL). No complications or serious adverse events were reported.

## Discussion

This study was designed to investigate which enhanced laparoscopic imaging techniques would show increased sensitivity and non-inferior specificity for the detection of peritoneal endometriosis. The use of 3D white-light imaging significantly improved the sensitivity rates for the detection of endometriotic lesions when compared to conventional 2D white-light imaging, and showed a comparable specificity rate. The combined use of NBI and 3D white-light imaging, both available on the standard laparoscopic platform, resulted in the highest sensitivity rate for the detection of peritoneal endometriotic lesions. The use of NBI and NIR-ICG alone showed to be of less value with decreased specificity and sensitivity rates, respectively.

These are novel findings with potential to improve surgical care for patients with endometriosis and were not reported in previous studies on the use of enhanced laparoscopic imaging techniques in endometriosis surgery. Additional analyses accounting for clustering of lesions within patients did not change our results and conclusions.

Previous studies in other fields of surgery showed that experienced surgeons’ skills improve with 3D vision compared to 2D vision [31, 32]. It is hypothesized that a 3D view of endometriotic lesions may be advantageous for the identification of endometriosis detection, showing more clearly an altered peritoneal lining and thickening induced by endometriosis. Inflammation causes a slight swelling of the peritoneum which presumably is the base of this improved recognition by 3D view.

In our clinical study, a (indirect) combination of NBI and 3D white-light imaging resulted in a significantly improved sensitivity rate. The application and addition of NBI to 3D white-light imaging is useful since the visualization of micro-vascularization is enhanced, as is observed in endoscopy for adenomatous lesions [33]. In addition, since NBI is integrated in the standard imaging processor of 2D and 3D equipment, no additional material or handling is necessary to incorporate these imaging techniques together in daily practice. Previous studies investigating the use of NBI [20, 21] also showed an increased sensitivity rate when NBI was added to 2D white-light imaging for the identification of peritoneal endometriosis. However, the use of NBI alone was not superior compared to the combined 2D/NBI modality and no control biopsies were taken to calculate specificity rates. The low specificity rate of NBI (with and without combined 3D view) of 71% may lead to an unjustified resection of presumed endometriotic lesions and a subsequent increased risk for surgical complications. However, since the excised endometriotic lesions are small and in need for small laser ablative therapy, with low associated morbidity, this might be of less clinical relevance. To increase specificity rates in the future, a learning phase with direct feedback of each nodule after reviewing the pathology might increase the specificity without hampering sensitivity rates.

Two case reports have been published regarding the use of NIR-ICG [22, 23]. In both studies, Firefly fluorescence detection systems (Intuitive Surgical Inc., Sunnyvale, USA) were used. The authors describe fluorescence has aided them in detection and resection of endometriosis. A recent cohort study was published, comparing NIR-ICG to 2D white-light imaging, revealing comparable sensitivity and specificity rates [34]. In our trial, however, a significantly decreased sensitivity rate of 36% compared to 2D white-light imaging was detected. Pathologic examination revealed that 43 out of 66 endometriotic lesions were not identified by fluorescent imaging. In our study, we administered the ICG directly under near-infrared vision, but up to 5 min was utilized to detect endometriotic lesions. Cosentino et al. (2018) administered ICG 5–30 min prior to inspection. Based on their results, we believe the use of NIR-ICG for detection of endometriosis could be further examined. However, most promising field of fluorescence-guided surgery will probably be the use of monoclonal endometriosis-specific antibodies coupled to a fluorescent marker. These potential promising new strategies deserves further research, as has been shown for cancer detection with CEA (bevacizumab) labeled to IRdye-800CW (a fluorescent agent, comparable to ICG) [35].

In this study, we decided not to investigate the 5-ALA technique [14–16]. Due to increased photosensitivity of the skin, patients are required to refrain from light for 24 h after ingestion of 5-ALA. We believe this is not patient friendly and not feasible for our patients whom are treated in day surgery. Secondly, the use of 5-ALA would not be cost effective.

Intra-operative identification of endometriosis is not only depending upon the ability to visualize lesions, but also upon the experience of the surgeon who performs the inspection. In this study, all procedures were performed by the same gynecologist (VM), an experienced endometriosis specialist. A potential weakness is the bias presented by the use of a single surgeon for all imaging modalities. Therefore, we have randomized the order in which imaging techniques were applied during laparoscopic inspection. Moreover, we have blinded the pathologist for the method of detection and clinical suspicion of endometriosis, in order to minimize bias and strengthen the results of this study. This trial was limited by the fact that the interrater variability was not assessed, neither was the learning curve. Although the surgeon had a large experience in detection of endometriosis with 2D white-light imaging, this was less for 3D white imaging, NBI, and NIR-ICG. Potentially after a learning curve, even better performance could be present with these imaging techniques. Further validation of the different imaging techniques should be addressed in future studies.

In this clinical trial, only patients with preoperatively established moderate to severe endometriosis (ASRM III–IV) were included, as we expected to encounter a sufficient amount of peritoneal disease in them. This study does not answer the question whether enhanced imaging techniques improve the detection of endometriosis in patients with limited peritoneal disease (ASRM I–II). In patients with limited peritoneal disease, an increased specificity (i.e., improved accuracy in showing the absence of disease) might be preferable, whereas in patients with high disease load (ASRM III–IV) improved sensitivity is preferable in order to achieve complete resection.

This study was designed as an explorative development study to identify which enhanced laparoscopic imaging technique has the best diagnostic accuracy for the detection of peritoneal endometriosis in terms of sensitivity (superior) and specificity (non-inferior). Based on the results of this trial, a further exploration of the use of NBI/3D white-light imaging is needed and will now be further evaluated in a large randomized clinical trial with clinical relevant endpoints according to the IDEAL framework with adequate power, quality control, and measures [36]. The clinical question is whether an improved detection of endometriosis with NBI/3D imaging also affects the long-term clinical outcomes after surgery, like reintervention rates, pain-free interval, and quality of life.

## Conclusions

Enhanced laparoscopic imaging with 3D white light, combined with NBI, improves the detection rate of peritoneal endometriosis when compared to conventional 2D white-light imaging. The use of these imaging techniques may potentially result in a more complete laparoscopic resection of endometriosis.
